# The UPS: a promising target for breast cancer treatment

**DOI:** 10.1186/1471-2091-9-S1-S2

**Published:** 2008-10-21

**Authors:** Ko Sato, Eeson Rajendra, Tomohiko Ohta

**Affiliations:** 1 Division of Breast and Endocrine Surgery, St Marianna University School of Medicine, Kawasaki, 216-8511, Japan; 2 Hutchison/MRC Research Centre, Cambridge, CB2 0XZ, UK

## Abstract

During the past decade, progress in endocrine therapy and the use of trastuzumab has significantly contributed to the decline in breast cancer mortality for hormone receptor-positive and ERBB2 (HER2)-positive cases, respectively. As a result of these advances, a breast cancer cluster with poor prognosis that is negative for the estrogen receptor (ESR1), the progesterone receptor (PRGR) and ERBB2 (triple negative) has come to the forefront of medical therapeutic attention. DNA microarray analyses have revealed that this cluster is phenotypically most like the basal-like breast cancer that is caused by deficiencies in the BRCA1 pathways. To gain further improvements in breast cancer survival, new types of drugs might be required, and small molecules targeting the ubiquitin proteasome system have moved into the spotlight. The success of bortezomib in the treatment of multiple myeloma has sent encouraging signals that proteasome inhibitors could be used to treat other types of cancers. In addition, ubiquitin E3s involved in ESR1, ERBB2 or BRCA1 pathways could be ideal targets for therapeutic intervention. This review summarizes the ubiquitin proteasome pathways related to these proteins and discusses the possibility of new drugs for the treatment of breast cancers.

Republished from Current BioData's Targeted Proteins database (TPdb; ).

## Protein pathway involvement in disease

### The UPS in breast cancer

The ubiquitin proteasome system (UPS) consists of several crucial enzymes: a ubiquitin-activating enzyme (E1), a ubiquitin-conjugating enzyme (E2), a ubiquitin ligase (E3) and the 26S proteasome [1,2]. The E3 catalyzes the formation of polyubiquitin chains (and sometimes monoubiquitylation), utilizing ubiquitin monomers that have been activated by the E1 and E2 enzymes, and transfers them onto a specific substrate(s). Depending upon the type of ubiquitin chain, the ubiquitin modifications signal a variety of processes, including 26S proteasome-dependent degradation [3-5]. By contrast, ubiquitin modifications are negatively controlled by deubiquitylation enzymes (DUBs) [6].

Remarkably, there are ~1000 E3s, categorized into subfamilies based on the structure of their catalytic site, including 300–500 Cullin-ROC/Rbx complexes, ~450 RING-type proteins, ~40 HECT-type proteins and ~20 U-box-type proteins. When comparing this with the ~500 mammalian protein kinases, it is easy to appreciate that the UPS contributes to most, if not all, cellular events. Therefore, it is realistic to anticipate major drug discoveries from this field, just like there have been in the kinase arena. Indeed, startling breakthroughs have been achieved recently with proteasome inhibitors.

### Estrogen receptors and the UPS

The α subunit of the estrogen receptor (ESR1) is degraded by the UPS, and compounds inhibiting its degradation could accelerate breast cancer growth [7]. However, the mechanism underlying its proteolysis might not be straightforward as there are at least two pathways for degradation: ligand-independent and ligand-dependent. For ligand-independent degradation, unliganded ESR1 associates with a protein complex containing Hsc/Hsp70 (a protein chaperone) and STUB1 (CHIP), an E3 ligase containing a U-box. STUB1 preferentially recognizes misfolded ESR1 and targets this protein for ubiquitin-mediated proteolysis. This pathway is important for the quality control of ESR1 [8,9]. Inhibition of this pathway could increase the active ESR1 pool. Alternatively, a dominant-negative effect could be induced by accumulation of misfolded ESR1.

Ligand-dependent degradation of ESR1 is mediated by different E3 ligases and is required for estrogen-induced transactivation. In HeLa cells expressing ESR1, treatment with MG132, a proteasome inhibitor, resulted in ESR1 stabilization but impaired ESR1-mediated transcription [10]. Cyclical recruitment of E3 ligases to ESR1 and binding of ESR1 to the proteasome is necessary for transcriptional activation of estrogen-responsive promoters [11,12]. During this process, the proteasome plays a central role in the clearance of ESR1-regulated transcription complexes, and inhibition of proteasomal activity prevents cycling of ESR1 onto promoters. Putative proteins involved in this process include: i) E3 ligases UBE3A (E6AP) [13,14], MDM2 [15] and TRI25 (EFP) [16]; ii) the 20S catalytic proteasome subunit beta type-9 (PSB9; also known as LMP2) [12] and the 26S protease regulatory subunit 8 (PRS8; also known as Rpt6/TRIP1/SUG1), which is a subunit of the 19S regulatory cap of the proteasome [11]; and iii) NCOA1 (SRC), which interacts directly with PSB9 [12] and NCOA3 (AIB1), which interacts with UBE3A [17]. UBE3A and TRI25 are preferentially recruited to estrogen-liganded ESR1, whereas MDM2 preferentially, but not exclusively, associates with unliganded ESR1 [11,16]. NCOA3 is the p160 ESR1 transcriptional coactivator and is amplified or overexpressed in breast cancer. An *Ncoa3 *transgenic mouse shows a high incidence of tumors in multiple organs, including breast [18,19].

### ERBB2, EGFR and UPS

ERBB2 (HER2) and EGFR (ERBB1, HER1) are growth factor receptors (GFRs), members of the transmembrane receptor tyrosine kinase family, and are overexpressed in 25–30% and 7–20% of breast cancers, respectively [20-22]. Similar to ESR1 ligand-dependent and -independent degradation, ubiquitylation is involved in the downregulation of GFRs. Ligand-dependent dimerization of EGFR increases tyrosine kinase activity and autophosphorylation of cytoplasmic tyrosine residues [23], and this enables interaction with the RING-domain-containing E3 ubiquitin-protein ligase CBL (c-CBL) in addition to downstream effectors SHC1 (SHC) and GRB2. CBL is phosphorylated by EGFR, which increases its ubiquitin ligase activity towards EGFR [24]. EGFR is then translocated to the endosomal compartment by SH3K1 (CIN85) and endophilins [25] and subsequently sorted for either recycling to the plasma membrane or destruction [26,27]. By contrast, phosphorylated ERBB2 only weakly associates with CBL and is resistant to CBL-induced downregulation [28]. In addition, ERBB2-EGFR heterodimerization blocks CBL association with EGFR and inhibits its lysosomal degradation, resulting in enhanced signaling [28-31]. Interestingly, antibody-induced ERBB2 tyrosine phosphorylation at Tyr1112 mediates interaction of the receptor with CBL, leading to receptor ubiquitylation and degradation [32]. By contrast, ligand-independent degradation of ERBB2 is mediated by STUB1 in collaboration with heatshock proteins Hsp70 and Hsp90 [33-36].

### BRCA1 and basal-like cancer

Gene-expression profiling identified basal-like breast cancer as an exceptional cluster with poor prognosis and a unique chemosensitivity. These cancers express basal/myoepithelial cell markers such as cytokeratins 5/6, 14 and 17 or vimentin [37-39] but do not express ESR1, progesterone receptor (PRGR) or ERBB2 (triple negative) [37]. Approximately 15% of sporadic breast cancers are characterized by this phenotype [37], but this particular cluster additionally possesses the dominant characteristics of aggressive breast cancers that are insensitive to both hormone therapy and trastuzumab (Herceptin, Genentech) [38], a humanized monoclonal antibody directed against ERBB2. Therefore, treatments that specifically target this subset of breast cancers could dramatically improve the overall prognosis for breast cancers. Interestingly, 80–90% of hereditary breast cancers with mutations in the gene encoding BRCA1 display a basal-like phenotype, suggesting that a deficiency in the BRCA1 pathway might cause this specific phenotype [39-43]. Indeed, BRCA1 dysfunction in sporadic basal-like cancers has been reported [44-46], and conditional deletion of exons encoding the C-terminus of BRCA1 in the mammary gland of mice results in basal-like cancer [47]. Thus, investigating the BRCA1 pathway could be an important approach for the treatment of basal-like cancer.

**Figure 1:**
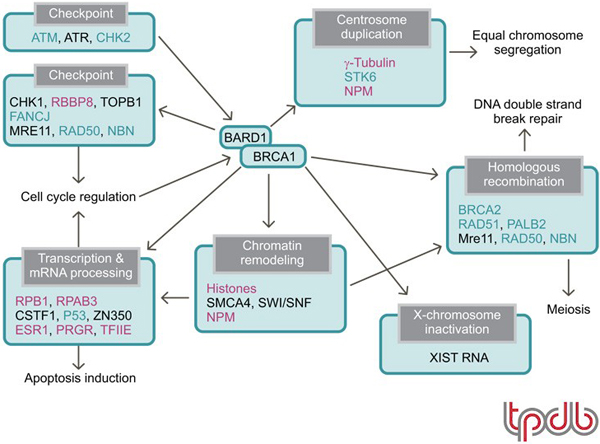
**Functional network of BRCA1 and its interacting proteins that maintains genomic stability**. Mauve: putative substrates for the BRCA1-BARD1 E3 ligase. Aqua: proteins encoded by genes that cause familial breast cancer or increase the risk of getting breast cancer. Please note that Swiss-Prot entry name synonyms have been used in figure – common synonyms are given in brackets: CHK2 (Chk2); CHK1 (Chk1); RBBP8 (CtIP); TOPB1 (TopBP1); FANCJ (BACH1/BRIP1); MRE11 (Mre11); RAD50 (Rad50); NBN (Nbs1); RPB1 (Pol II [RPB1]); RPAB3 (RPB8); CSTF1 (CstF50); P53 (p53); ZN350 (ZBRK1); ESR1 (ER-α); PRGR (PR); STK6 (Aurora A); NPM (NPM1/B23); BRCA2 (FANCD1); RAD51 (Rad51); PALB2 (FANCN); SMCA4 (BRG1).

BRCA1 acts as a hub protein that coordinates a diverse range of cellular pathways to maintain genomic stability [48]. Figure 1 shows some representative functions of BRCA1. Many proteins whose mutation is implicated in familial breast cancer, such as serine-protein kinase ATM [49,50] and serine/threonine-protein kinase CHK2 [51-53], are involved in this functional network. Mutation of the central *BRCA1 *gene results in ~80% penetrance of breast cancer, and *BRCA1 *gene methylation or the accumulated dysfunctions of other proteins whose single mutation causes low penetrance are thought to result in sporadic breast cancer [48,54]. BRCA1 is a component of several different super-complexes, and, importantly, BRCA1 partners with BARD1 to form a RING heterodimer ubiquitin ligase [55,56] in most of these complexes [57]. This suggests that the BRCA1-BARD1 complex directs the ubiquitylation of distinct substrates within each complex. BRCA1-BARD1 catalyzes the formation of unconventional polyubiquitin chains that include Lys6-linked chains [58-60] or catalyzes the monoubiquitylation of some substrates [61-64]. The putative substrates of BRCA1 in each complex are shown in Figure 1. Histones [61,62], γ-tubulin [63] and ESR1 [64] are monoubiquitylated, whereas NPM1 [65], RPB1 [66,67], RBBP8 (CtIP) [68], RPAB3 (RPB8) [69], PRGR [70] and T2EA/T2EB (also known as general transcription factor IIE or TFIIE) [71] are polyubiquitylated and/or multiubiquitylated. Phosphorylated RPB1 and PRGR are ubiquitylated and degraded *in vivo *in the presence of BRCA1 [66,70]. However, there is presently no direct evidence supporting the notion that BRCA1-mediated ubiquitylation signals degradation. For the other polyubiquitylated substrates NPM1 [72], RBBP8 [68], RPAB3 [69] and T2EA/T2EB [71], as well as for BRCA1 autoubiquitylation [59], it has been proposed that the ubiquitylation is not a signal for degradation. Although the biochemical mechanism regarding how the ubiquitin modifications affect intermolecular functions remains to be clarified, some biological consequences of the modifications have been reported. RBBP8 ubiquitylation depends on the phosphorylation-mediated interaction between RBBP8 and BRCA1 BRCT domains (a phosphoserine/threonine binding motif), and ubiquitylated RBBP8 associates with chromatin after DNA damage to participate in G2/M checkpoint control [68]. RPAB3 is polyubiquitylated by BRCA1 after UV irradiation, and HeLa cells expressing a ubiquitin-resistant form of RPAB3 exhibit UV hypersensitivity [69]. BRCA1-mediated ubiquitylation of the T2EA subunit of T2EA/T2EB blocks the initiation of mRNA synthesis by preventing the association between the pre-initiation complex and both TFIIE and the general transcription factor IIH (TFIIH) [71].

## Disease models, knockouts and assays

Basal-like cancer most closely resembles features of hereditary breast cancer associated with *BRCA1 *mutation. It also displays frequent mutations in the *TP53 *gene (encoding cellular tumor antigen P53). Based on this correlation, conditional mouse mutants with somatic deletion of *Brca1 *and *Tp53 *in mammary epithelium have been generated. Female mice of this strain show a high incidence of mammary tumors. Furthermore, the phenotype of these tumors mimics many aspects of human basal-like breast cancer [73]. More specifically, conditional mouse mutants with a deletion of the C-terminus of *Brca1 *and also heterozygous for a *Tp53 *mutation have also been generated. These show a high incidence of breast tumors that are similar to human basal-like cancer. [47] These mouse models could well prove useful for the study of basal-like cancer treatments.

It has recently been established that chimeric proteins containing the tyrosine kinase and RING domain of CBL and substituted Src-homology 2 domains from GRB2, from the P85 regulatory subunits of phosphoinositide 3-kinase (PI3K) or from SRC were capable of mediating ubiquitin-dependent proteolysis of ERBB2 (HER2) [74]. Although no physiological E3 ligases for the estrogen receptor (ESR1) and ERBB2 ubiquitylation have yet been identified, this model could prove useful for understanding ERBB2-positive cancer. Despite the fact that there are no mouse models of ERBB2 and ESR1 function in breast cancer, there is good potential for novel models to be generated for dissecting the roles of these two proteins.

## Disease targets and ligands

### The UPS and proteasome inhibitors

The first proteasome inhibitor that has come into clinical practice is bortezomib (Velcade, PS-341, Millennium) [75]. Approximately a third of relapsed, refractory multiple myeloma patients show a significant response to bortezomib [76], and the US FDA approved bortezomib for use as a therapy for multiple myeloma in 2003. Bortezomib inhibits proteasome function in a slowly reversible manner by means of an interaction between boronic acid at the C-terminus of bortezomib and an active threonine in the chymotryptic catalytic site of the 20S proteasome [77]. The mechanisms underlying the therapeutic effect of bortezomib in multiple myeloma have been investigated intensively. Inhibition of the transcription factor NFκB by blocking the degradation of its inhibitory partner IκB is one such putative model. However, recent studies suggest that multiple factors might contribute to the efficacy of the drug [78]. An interesting recent study showed that inhibition of the 26S proteasome by MG132 causes a depletion of available nuclear ubiquitin because of the accumulation of nondegraded polyubiquitylated proteins in the cytosol. The depletion of free nuclear ubiquitin resulted in a loss of monoubiquitylated histones, and consequently this might have impaired many nuclear regulatory systems, including the DNA-damage responses [79,80].

Clinical trials of bortezomib in many hematologic (e.g. a phase II trial on cutaneous T-cell lymphoma by Jonsson Comprehensive Cancer Center, NCT00182637) and non-hematologic malignancies (e.g. phase II trials on advanced bronchiolo-aveolar carcinoma [BAC] or adenocarcinoma by the California Cancer Consortium, NCT00118144, and on pleural mesothelioma by the Irish Clinical Oncology Research Group, NCT00513877) are ongoing. Thus far, bortezomib has failed to show a significant clinical effect on breast cancer. Although bortezomib was well tolerated, no responses were observed in 12 patients with aggressive metastatic breast cancers, with extremely poor prognoses, when used as a single agent [81]. However, the effect of combination therapy and the therapeutic effect for selected patients, such as those with tumors expressing a particular hormone receptor, ERBB2 (HER2) status or those in earlier stages of breast cancer, remain to be determined.

There are increasing numbers of small molecules that target the UPS. The UPS Patent Portfolio table and UPS Drugs & Biologicals table on the *Targeted Proteins database *show the patents and drugs in development or on the market (and the associated organizations and companies) designed to inhibit the UPS that might be useful in breast cancer therapy. An orally active proteasome inhibitor salinosporamide A (NPI0052, Nereus), a natural product derived from a marine actinomycete, resembles lactacystin and irreversibly targets the proteasome [82,83,72]. Epoxomicin and eponemicin are epoxyketone-containing natural products that exhibit antitumor activity [84,85]. Epoxomicin, currently the most specific proteasome inhibitor, reacts irreversibly with the chymotrypsin-like site, whereas the less-potent epoxyketone eponemicin reacts with both the caspase-like and chymotrypsin-like sites of the proteasome [86-88]. PR171 (carfilzomib, Proteolix), a synthetic derivative of epoxomicin, has been shown recently to have antiproliferative and proapoptotic effects on primary human acute myeloid leukemia cells [89].

### Estrogen receptors and the UPS

Many anti-breast cancer drugs are involved in degradation of the estrogen receptor (ESR1) [90]. The pure antagonist fulvestrant (ICI 182780, AstraZeneca) and RU58668 promote degradation of ESR1, whereas 4-hydroxy-tamoxifen does not [91]. Fulvestrant removes ESR1 from cyclic recruitment to its target promoters by direct targeting of ESR1 to the proteasome without associated transcription [11]. NCOA3 (AIB1) also induces agonist-induced, but not antagonist-induced, degradation of ESR1. Therefore, the suppression of NCOA3 that results in inhibition of estrogen-induced degradation of ESR1, but not degradation induced by fulvestrant or GW5638 [17], could have additional clinical effects for ESR1-positive breast cancer. Other compounds that induce degradation of ESR1 include 2,3,7,8-tetrachlorodibenzo-p-dioxin (TCDD), a ligand of the aryl hydrocarbon receptor (AHR) [92], and PPARG agonists ciglitazone and 15-deoxy-delta 12,14-prostaglandin J2 [93].

Although these existing drugs have not been designed to modify the UPS, it is possible that they function primarily through UPS modulation. In this regard, revealing the precise mechanisms of degradation of ESR1 with respect to the UPS might further improve the effectiveness of such compounds for the treatment of breast cancer. Compounds that affect the interactions of E3 ligases, coactivators and proteasome subunits in ESR1-regulated complexes on promoters could work as anti-breast cancer agents.

### ERBB2, EGFR and the UPS

As mentioned previously, ligand-independent degradation of ERBB2 is mediated by STUB1 (CHIP) in collaboration with Hsp70 and Hsp90 [33-36]. Therefore, acceleration of this pathway might have additive anticancer activity when introduced with trastuzumab. The potent anticancer agent geldanamycin, a benzoquinone ansamycin that binds to Hsp90, is one such candidate [33,34]. In addition, the STUB1-dependent degradation pathway of ERBB2 can be stimulated by tyrosine kinase inhibitors such as CI1033 (Pfizer) [35]. CI1033 and geldanamycin additively inhibit tumor cell growth.

Thus, downregulation of ERBB2 by means of acceleration of the UPS is of crucial importance to breast cancer treatment. Interestingly, however, the proteasome inhibitor bortezomib has an additive or synergistic effect with trastuzumab in the induction of apoptosis in ERBB2-positive breast cancer cell lines [94]. It is likely that inhibition of other UPS pathways contributes to this effect. Alternatively, it could be caused by a depletion of available nuclear ubiquitin due to the accumulation of nondegraded polyubiquitinated proteins, as mentioned previously [79,80].

Targets downstream of ERBB2 could also be affected by the UPS. BIRC5 (also known as survivin), an anti-apoptotic protein, is expressed only in tumors. Expression of BIRC5 is associated with a poor prognosis in a variety of malignancies, including breast cancer [95,96]. Recent studies revealed that BIRC5 is regulated by ERBB2 and ERBB3 but not by EGFR [97]. Interrupting the ERBB2-ERBB3 heterodimer using lapatinib (GW572016, GlaxoSmithKline), a potent small-molecule inhibitor of EGFR and ERBB2 tyrosine kinases, leads to proteasomal degradation of BIRC5 and induces apoptosis both in breast cancer cell lines overexpressing ERBB2 and in primary tumors [97]. Understanding the mechanisms that protect ERBB2-overexpressing cancer cells from apoptosis might result in new targets for therapeutic intervention.

## New frontiers in drug discovery

The therapeutic effect of proteasome inhibitors on breast cancer remains to be determined but is greatly anticipated. Additionally, interest is focused on the discovery of other potent anticancer drugs that affect substrate ubiquitylation by E3 ligases as well as their deubiquitylation catalyzed by DUB enzymes. Because inhibition of the proteasome, whose activity is crucial for all cell types, imparts such a specific clinical effect, inhibiting the catalytic site of E3 ligases that regulate a broad range of cellular processes, for example the RBX1 (ROC1) RING finger subunit of Cullin-based E3 complexes [98,99], could be equally valuable. Alternatively, E3 ligases found to be oncogenes or tumor suppressor genes that regulate more restricted pathways could be promising targets for small-molecule inhibitors and activators, respectively, with fewer side effects [100]. The following sections describe such specific pathways that link directly to the breast cancer clinic.

### Estrogen receptors and the UPS

Progress in hormone therapy, including aromatase inhibitors and the gonadotrophin-releasing hormone agonist goserelin, has been a major contribution to the recent improvements in breast cancer prognosis. As there already exist several effective hormone therapy agents, it might be conjectured that discovering additional drugs for the same pathway is not necessary. However, approximately half of hormone receptor-positive breast cancers do not respond sufficiently to the current hormone therapies [101], and therefore alternative drugs free from cross-tolerance are needed. Compounds targeting the UPS in hormone receptor signaling could be one such alternative.

### ERBB2, EGFR and UPS

Trastuzumab prolongs the survival of patients with metastatic ERBB2 (HER2)-positive breast cancer and leads to dramatic improvements in prognosis when used in adjuvant therapy [102]. Indeed, trastuzumab has switched ERBB2-positive breast cancer from being a subset with the worst prognosis to one that is curable with existing therapy [102]. However, even in a subset of patients with highly ERBB2-positive tumors, the response rate to trastuzumab is still limited, and alternative compounds that target the ERBB2 pathway could have additive or synergistic roles. The UPS is definitely one such candidate. In addition to ERBB2, EGFR is overexpressed in basal-like breast cancer and is a possible target for new anti-breast cancer reagents.

### BRCA1 and basal-like cancer

Although the significance of the E3 activity of BRCA1 in the BRCA1 functional network (Figure 1) is only partially understood, it is obvious that its activity is crucial in the prevention of a certain subset of breast cancers [56,103-105]. In terms of the potential for targeting this activity for breast cancer therapy, tumor suppressors such as BRCA1 are not ideal targets for therapy because small molecule activators can be more difficult to produce than small molecule inhibitors. This is absolutely true for the treatment of breast cancer caused by *BRCA1 *gene mutations. However, if the breast cancer results from other factors that cause downregulation of BRCA1 – for instance, in the case of sporadic basal-like breast cancer – small molecules that activate BRCA1 E3 activity could be successful.

## List of abbreviations used

DUB: deubiquitylation enzyme; GFR: growth factor receptor; PI3K: phosphoinositide 3-kinase; UPS: ubiquitin proteasome system.

## Competing interests

T. Ohta is the author of two patents:

1) Ohta T. **Carcinostatic method using BRCA1-BARD1 pathway**. WO2005073379 (08/11/2005).

2) Ohta T and Wu W. **A method for the ubiquitination of common subunits of RNA polymerases**. WO2007046538 (04/26/2007).

## Publication history

Republished from Current BioData's Targeted Proteins database (TPdb; ).
